# Efficient labeling *in vitro* with non-ionic gadolinium magnetic resonance imaging contrast agent and fluorescent transfection agent in bone marrow stromal cells of neonatal rats

**DOI:** 10.3892/mmr.2015.3532

**Published:** 2015-03-23

**Authors:** YING-QIN LI, YING TANG, RAO FU, QIU-HUA MENG, XUE ZHOU, ZE-MIN LING, XIAO CHENG, SU-WEI TIAN, GUO-JIE WANG, XUE-GUO LIU, LI-HUA ZHOU

**Affiliations:** 1 Department of Anatomy, Zhong Shan School of Medicine, Sun Yat-sen University, Guangzhou, Guangdong 510080, P.R. China; 2 Department of Radiology, The Fifth Affiliated Hospital of Sun Yat-sen University, Zhuhai, Guangdong 519000, P.R. China; 3 Department of Radiology, The First Affiliated Hospital of Guangzhou Medical University, Guangzhou, Guangdong 510120, P.R. China; 4 Department of Anesthesiology, The First Affiliated Hospital of Sun Yat-sen University, Guangzhou, Guangdong 510080, P.R. China; 5 Department of Encephalopathy Center, Guangdong Provincial Hospital of Traditional Chinese Medicine, Guangzhou, Guangdong 510120, P.R. China

**Keywords:** gadodiamide, magnetic resonance imaging, bone marrow stromal cells

## Abstract

Although studies have been undertaken on gadolinium labeling-based molecular imaging in magnetic resonance imaging (MRI), the use of non-ionic gadolinium in the tracking of stem cells remains uncommon. To investigate the efficiency in tracking of stem cells with non-ionic gadolinium as an MRI contrast agent, a rhodamine-conjugated fluorescent reagent was used to label bone marrow stromal cells (BMSCs) of neonatal rats *in vitro*, and MRI scanning was undertaken. The fluorescent-conjugated cell uptake reagents were able to deliver gadodiamide into BMSCs, and cell uptake was verified using flow cytometry. In addition, the labeled stem cells with paramagnetic contrast medium remained detectable by an MRI monitor for a minimum of 28 days. The present study suggested that this method can be applied efficiently and safely for the labeling and tracking of bone marrow stromal cells in neonatal rats.

## Introduction

Stem cell therapy has the potential to improve the treatment of patients with various diseases. Bone marrow stromal cells (BMSCs) possess various characteristics, including multi-directional differentiation, promotion of stem cell implantation, hematopoietic support, immune regulation and self-regulation (1–4). Furthermore, under specific induced conditions, BMSCs are able to differentiate into various types of cell, including adipocytes, osteocytes, chondrocytes and hematopoietic cells, as well as various types of tissue, including muscle, nerve, endothelium, liver and myocardium (5–10). Therefore, BMSCs are considered to be ideal cells for stem cell therapy (11–16).

In addition to the progression of stem cell transplantation (SCT), its tracking technology has been demonstrated to be important. Conventional methods used for stem cell detection are invasive, which require animal organs and tissues for histological microtome sections and immunochemical examinations (17). Medical imaging has progressed from traditional methods using anatomical morphological analysis, to current molecular imaging (18) which is based on cellular, genetic and molecular information in addition to signaling pathways. With the progress of imaging and SCT, SCT has become an international research focus (19). Magnetic resonance imaging (MRI) has certain advantages over computer tomography (CT) and nuclear medicine in molecular imaging, including high temporal resolution, satisfactory tissue contrast ratio, long evaluation time and absence of radiation pollution (20,21). Previous studies on inflammation (22), cancer (23), immune reactions and the efficacy of stem cell treatment have demonstrated that MRI is able to detect the dynamic migration of stem cells *in vivo* (24,25). Thus, the use of MRI is suggested to be beneficial to molecular medicine. Currently, stem cell labeling and tracking using MRI contrast agents is a focus of research due to its potential to improve MRI visualization *in vivo*. Gadodiamide, also known as Omniscan, is a positive MRI contrast agent and a clinically safe gadolinium-chelate complex, which is injected as a parenteral non-ionic gadolinium solution (26,27). Gadodiamide is a chelate compound containing non-ionic pentetic acid and gadolinium, and its chemical formula is [5,8-bis(carboxymethyl)-11-[2-(methylam ino)-2-oxoethyl]-3-oxo-2,5,8,11-tetraazatridecan-13-oato(3-)] gadolinium (C_16_H_26_GdN_5_O_8_).

In order to explore a novel and feasible molecular target, the present study delivered gadodiamide into the BMSCs of neonatal rats and assessed its feasibility, safety and efficacy. In addition, clinical MRI signal characteristics of BMSC labeling *in vitro* were analyzed for further investigation *in vivo*.

## Materials and methods

### Animals

A total of 45 female neonatal Sprague-Dawley (SD) rats (9-11 days old; 15–20 g) used in this experiment were obtained from the Experimental Animal Center of Sun Yat-sen University (Guangzhou, China) (animal use permit no. SYXK 2012-0081). The experiments in the present study were conducted aseptically in accordance with the Chinese National Health and Medical Research Council’s animal ethics guidelines. The use of these animals was approved by the Medical Ethics Committee of the 5th Affiliated Hospital of Sun Yat-sen University (Zhuhai, China).

### Isolation, cultivation and identification of BMSCs

The isolation and cultivation of BMSCs from SD rats was conducted as previously described (28). Three neonatal rats were sacrificed by cervical dislocation and the left and right tibias, femurs and humeri were removed under aseptic conditions. The soft tissue around the bones was also removed, and both ends were cut off. Partial epiphyseal cartilage was retained, since it contained a certain number of stem cells. The marrow cavity was pierced using a 1-ml sterile syringe and rinsed repeatedly with cell culture solution, which contained 10% FBS (Gibco life Technologies, Carlsbad, CA, USA) and 1% penicillin/streptomycin antibiotics (Solarbio Science & Technology Co., Ltd., Beijing, China). The homogeneous bone marrow cell suspension was collected through a stainless steel screen filter (aperture 75 *μ*m, 200 mesh) into a beaker and seeded in a 75-cm^2^ culture flask (Corning-Costar, Corning, NY, USA) covered with poly-lysine (PLL; Sigma-Aldrich, St. Louis, MO, USA). Cells were incubated at 37°C with 5% CO_2_ and the medium was replaced after 3 h. Following incubation for 5–7 days, the cells were passaged and re-plated. To distinguish the cultured cells from hematopoietic stem cells and evaluate the purity, BMSCs at passage 2 were detected by fluorescence-activated cell sorting (BD FACSAria III; BD Biosciences, Franklin Lakes, NJ, USA). Briefly, BMSCs (5×10^5^) were re-suspended in 500 *μ*l of phosphate-buffered saline (PBS; Gibco Life Technologies). The cells were incubated in the dark at 4°C for at least 15 min and collected for analysis within 1 h. The suspensions were then incubated with monoclonal anti-rat-CD29-phycoerythrin (PE; 1:1,000; cat. no. 12-0291-81; eBioscience, Inc., San Diego, CA, USA), monoclonal anti-rat-CD90-PE (1:1,000; cat. no. 12-0900-81; eBioscience, Inc.) and monoclonal anti-rat-CD45-fluorescein isothiocyanate (FITC; 1:1,000, cat. no. 11-0461-80; eBioscience, Inc.).

### Cell-labeling protocol

#### Medical materials

A gadodiamide solution (Omniscan; molecular weight, 574 Da; 20 ml solution containing 5.74 g gadodiamide solid drug in sterile redistilled water) was purchased from GE Healthcare Life Sciences (Little Chalfont, UK). The cell labeling agent used in the present study was the fluorescent Arrest-In transfection reagent (Open Biosystems, GE Healthcare Bio-Sciences), which is a polyethylenimine-based lipopolymeric formulation (29). An orthogonal experiment was conducted to achieve the highest transfer efficiency and minimized toxicity. The orthogonal experiment was designed according to an initial factor orthogonal experimental table to test MTT value (n=3), with 1×10^5^ cells/ml seeded in a 96-well plate (Corning-Costar). Optimum conditions based on various levels of the test were then selected. Subsequently, a repeated verification test was conducted to further optimize conditions for both minimal cytotoxicity and the optimum up-take efficiency. In the present study, the optimal cell-uptake method was as following: FI reagent/Gd solution/Opti-MEM = 1:3:200, and the mixing time and incubation time were 30 min and 3 h, respectively.

#### Cell labeling

Cells were seeded at a density of 5×10^5^ cells/well in a six-well plate with 2 ml Dulbecco’s modified Eagle’s medium (DMEM; Gibco Life Technologies)/F12 [10% fetal bovine serum (FBS)] culture medium. A total of 10 *μ*l FI-Arrest In reagent and 30 *μ*l gadodiamide were respectively dissolved in 500 *μ*l opti-MEM (Gibco Life Technologies), incubated for 15–20 min at room temperature (RT; 25°C), and were mixed for 30 min to produce the labeling mixture. The mixture was then carefully and slowly added to the plate, and Opti-MEM was added to a total amount of 2 ml per well. Following incubation at 37°C and 5% CO_2_ for 3 h, the mixed liquid was replaced by 2 ml fresh DMEM/F12 medium containing 10% FBS.

### Evaluation of cell uptake efficiency

#### Flow cytometric detection of labeling ratio

Following a 24-h resting period after labeling, 5–7×10^5^ labeled cells were suspended in 500 *μ*l PBS. Taking an equal quantity of non-labeled cells as controls, the cell uptake efficiency was analyzed using a flow cytometer (BD FACSAria III).

#### Inverted fluorescence microscopy

The labeled cells incubated for 8 h in the six-well plates were fixed with 4% paraformaldehyde (Sigma-Aldrich). The cells were then counter-stained and incubated at RT with Hoechst 33258 (1 ml/well; Beyotime Institute of Biotechnology, Haimen, China) in the dark for 15 min. After removal of Hoechst 33258, the cells were washed three times with PBS. The stained cells were observed under an inverted fluorescence microscope (DMI4000B; Leica Microsystems, Oberkochen, Germany).

#### Transmission electron microscopy (TEM)

TEM was used to observe the intracellular Gd^3+^ distribution. Cells were suspended in culture medium in a 1.5 ml tube and centrifuged at 100 × g for 5 min. Following removal of the supernatant, 3% glutaraldehyde (Beyotime Institute of Biotechnology) was added to fix cells for 30 min. The cells were further centrifuged at 100 × g for 5 min. The cells were sectioned using a diamond knife (Diatome Ltd., Biel, Switzerland) in order to obtain ultrathin sections, and double staining was conducted using uranyl acetate (Guanghua Chemical Factory Co., Ltd., Guangong, China) and lead citrate (Guanghua Chemical Factory Co., Ltd.).

### Comparision of cellular characteristics

#### Growth curves and survival ratio

The labeled cells were vaccinated in a 25-cm^2^ culture flask (Corning-Costar), and incubated at 37°C with 5% CO_2_. A total of 15 *μ*l re-suspension liquid from labeled BMSCs was added to 4% trypan blue solution (Beyotime Institute of Biotechnology) at a 1:1 ratio. The inverted microscope (DM IL; Leica Microsystems) was used to count the total cells. As the blue-stained cells were the dead cells, the cell survival ratio was calculated as [1−(number of blue-stained cells/total number of cells)] ×100%. Every other day, survival tests were performed on cells from the experimental and the control group. Three samples per group were examined, and counting of each sample was repeated four times. The cell numbers were counted, respectively between days 0–14 in order to construct cell growth curves, and the cell survival ratio was calculated for 24, 48 and 72 h.

#### Viability and proliferation of cells

The MTT assay was used to analyze cell viability and proliferation. Following 3 h of labeling, the cell suspension with fresh medium was adjusted to a density of 1×10^5^ cells/ml and seeded into a 96-well plate coated with PLL at 100 *μ*l/well. The cell vability was measured at days 0–5 following labeling. A total of 20 *μ*l MTT (Sigma-Aldrich) liquid was added to each well, and the wells were subsequently incubated for 4 h. All the medium and MTT liquid was removed and 150 *μ*l dimethyl sulfoxide (Sigma-Aldrich) added into test wells. A microplate reader (Multiskan MK3; Thermo Fisher Scientific, Waltham, MA, USA) was used to measure the optical density values at a wavelength of 490 mm. Using unlabeled cells as the control, the ratio between labeled cells and the control group was taken as the proliferation rate.

### In vitro MRI of Gd-labeled BMSCs

#### Cells groups

Cells were re-suspended and exposed to different concentrations of BMSCs for different time periods. A total of 200 *μ*l/well of the suspension was added into the 96-well plates. Centrifugation was conducted at 200 ×g for 5 min at 20°C (Allegra X-15R; Beckman Coulter, Brea, CA, USA), and the plate was washed thoroughly with 0.01 M PBS in order to eliminate the gadodiamide that had not been taken up by the cells. Cells were covered with 100 *μ*l 1% agarose solution (Agar; Sigma-Aldrich), which solidified at RT, to immobilize them.

#### Experimental groups

To analyze the MRI signal intensity and durability of cell labeling (BMSCs + Gd/FI) at different time-points, experimental groups were exposed to Gd + FI for 1, 3, 7, 14, 21 and 28 days. Each group contained three samples and each sample contained 1×10^6^ cells. The control groups were as follows: Cells with gadodiamide but no FI-Arrest In agent at day 1 (BMSCs + Gd/non-FI), non-labeled cells (BMSCs + Agar) and empty control without BMSCs (Agar + PLL).

#### MRI scanning parameters

MRI scanning was performed using a clinical 1.5 Tesla MRI scanner (Philips, Amsterdam, Netherlands) with a dedicated animal somatic coil with a radial line of 14.5 x 8 cm. Samples were flatly and centrally placed in the coil and transverse scanning was conducted. The scanning parameters were performed as follows: Field of vision, 130 × 148 × 79 mm (FH × RL × AP); voxel size, 0.9 × 1.12 mm (FH × RL); number of signal averages, 2; layer thickness, 1.5 mm; and layer distance, 1.5 mm. FH represents foot-to-head diameter, on behalf of the longitudinal axial diameter; RL represents right-to-left diameter, on behalf of the transverse axial diameter; and AP represents anteroposterior diameter. T1 weighted image and spin echo sequences (T1WI SE) parameters were TR = 250 msec and TE = 15 msec. Each sample was scanned six times repeatedly. Image-analysis software (Intera Achieva Nova Dual 1.5T MRI; Philips) was used to obtain the signal intensity and signal-to-noise ratio (SNR). The software was used to gate the areas of interest at the bottom of the scanning specimens. The area was 15 mm^2^ with a diameter of 14 mm. T1WI signal intensity and noise at the same level acquired MRI SNR data.

#### Statistical analysis

The results were analyzed using SPSS software, version 17.0 (SPSS, Inc., Chicago, IL, USA). All values are expressed as the mean ± standard deviation. Variance analysis was used to compare differences and the paired t-test was used for data analysis between the experimental and control groups. P<0.05 was considered to indicate a statistically significant difference.

## Results

### Identification and morphological observation of BMSCs

**Figure 1:**
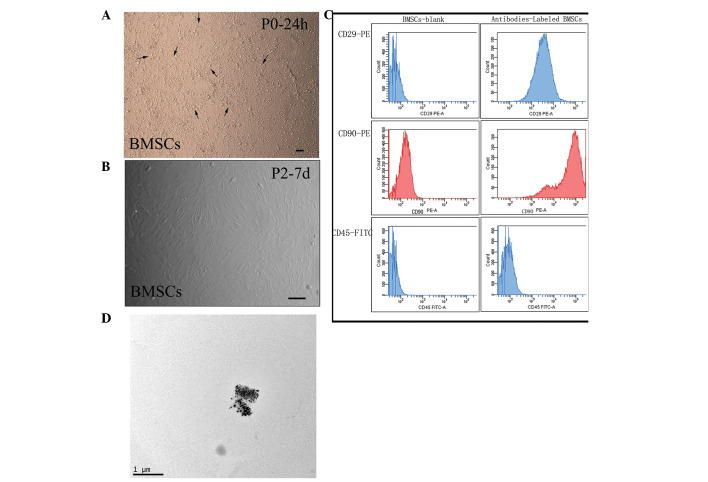
Morphology of cultured BMSCs and the physical characteristics of gadodiamide. (A and B) Morphology of cultured BMSCs. (A) Following 24-h of culture in Dulbecco’s modified Eagle’s medium/F12 medium, the number of adherent cells increased gradually and exhibited a spindle-shaped morphology, indicated by the black arrows. (B) At passage 2, the cells became homogeneous in morphology. (C) Fluoresnce-activated cell sorting analysis confirmed that the BMSCs homogeneously expressed specific surface antigens. The BMSCs were observed to be positive for CD29 and CD90, but negative for CD45. (D) Via transmission electron microscopy, gadodiamide was observed to be composed of black and dense gadolinium particles, of which the diameter was identified to be ~0.04 *μ*m. (A and B, scale bar = 100 *μ*m; D, scale bar = 1 *μ*m). BMSCs, bone marrow stromal cells; TEM, transmission electron microscopy.

In the experiment, following 24-h culture in DMEM/F12 medium, the number of adherent cells increased gradually and cells exhibited a spindle-shaped morphology (Fig. 1A). During 5–7 days, the primary BMSCs were confluent to 80–90% in a single layer. At passage 2, the cells became homogeneous in morphology (Fig. 1B). FACS analysis confirmed that the BMSCs homogeneously expressed specific surface antigens (Fig. 1C). They were observed to be positive for CD29 (95.83±3.35%) and CD90 (98.67±1.47%), but negative for CD45 (98.6±0.23%; n=3).

### TEM

According to TEM analysis, the diameter of the black and dense gadolinium particles was observed to be ~0.04 *μ*m (Fig. 1D).

### Cellular uptake and localization of gadodiamide

**Figure 2:**
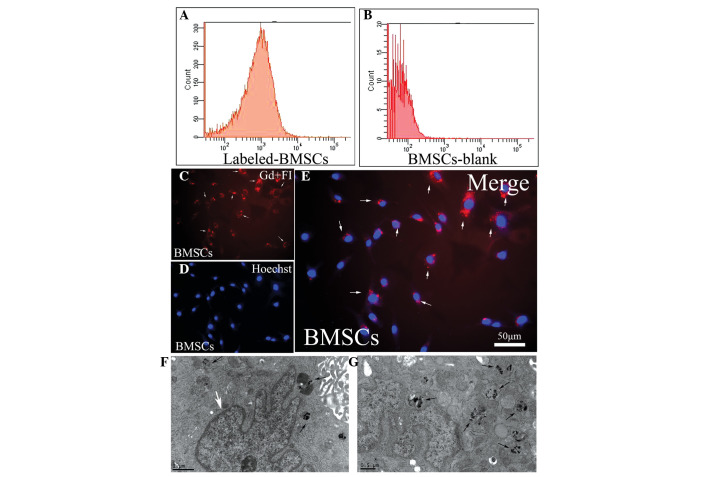
Evaluation of labeling efficiency with gadodiamide and FI-Arrest In reagent. (A and B) Flow cytometric analysis demonstrated the rhodamine-fluorescence ratio of (A) labeled BMSCs and (B) unlabeled cells as a control. (C–E) BMSCs labeled with gadodiamide for 3 h observed under an inverted fluorescence microscope. (C) The FI-Arrest In reagent combined with gadolinium is shown in red (white arrows) within the cytoplasm and (D) around the blue-stained cell nucleus. (E) Multiple cells were labeled with the complex as marked in the merged image (white arrows). (F and G) Transmission electron microscopy images showing that the black and dense gadolinium particles (black arrows) were clearly sporadic or confluent within the cytoplasm around the nuclei of labeled cells (white arrows). C–E, scale bar = 50 *μ*m; F, scale bar = 1 *μ*m; G, scale bar = 0.5 *μ*m. BMSCs, bone marrow stromal cells.

Flow cytometric analysis was used to detect the rhodamine-fluorescence ratio of gadodiamide-labeled cells (Fig. 2A) with unlabeled cells as a control (Fig. 2B). The positive labeling rate was 44.95±2.42% (n=3). As observed under an inverted fluorescence microscope, FI-Arrest In agent combined with Gd (red) was present within the cytoplasm and around the blue-stained cell nuclei (Fig. 2C–E). Under the transmission electron microscope, the black and dense gadolinium particles were clearly observed. They were sporadic or confluent within the cytoplasm of labeled cells (Fig. 2F and G); however, none were observed in the control group.

### Characteristics of labeled BMSCs

**Figure 3:**
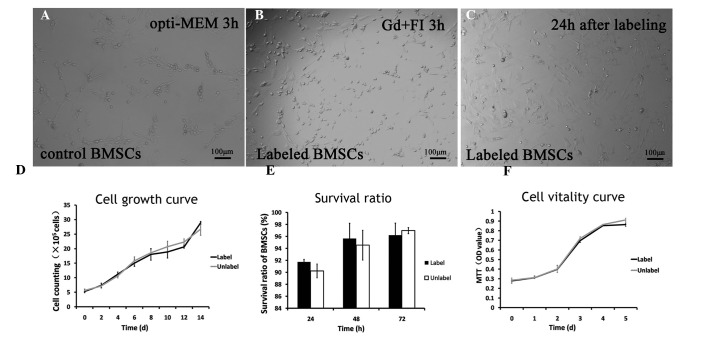
Cellular characteristics of BMSCs labeled with Gd/FI. BMSCs were observed under the inverted microscope (A) in the control group, (B) following labeling for 3 h and (C) following labeling for 24 h. No significant differences were observed between the labeled and unlabeled cells (P>0.05) in (D) the number of cells from 0–14 days following labeling, (E) the survival ratio at 24, 48 and 72 h and (F) the MTT-based cell viability 0–5 days subsequent to cell labeling. Scale bar in A–C = 100 *μ*m. BMSCs, bone marrow stromal cells; Gd/FI, gadodiamide/FI-Arrest In reagent; OD, optical density.

Following incubation for 3 h *in vitro*, BMSCs were observed under the inverted microscope. The cells in the experimental (labeled with Gd + FI for 3 h) and control (incubated with opti-MEM medium only) groups were observed to be similar in morphology and growth density (Fig. 3A–B). The cells were adherent to the wall of the culture flask and exhibited spindle-shaped morphology. Subsequently, the labeling solution was removed and DMEM/F12 containing 10% FBS was placed into a culture flask. Labeled cells were observed following incubation for 24 h (Fig. 3C). The adherent cells grew, and the number of cells was markedly increased.

Evaluation by trypan blue exclusion analysis identified no significant difference in the growth rate between the labeled and unlabeled BMSCs (P>0.05) from 0–14 days subsequent to cell labeling (Fig. 3D). The growth curve demonstrated that the total number of cells in the labeled and unlabeled groups increased in a time-dependent manner, particularly during 2–8 days following cell labeling. No significant difference was observed in the survival ratio between labeled cells and the control group at 24, 48 and 72 h (Fig. 3E). The MTT-based cell viability evaluation confirmed that optical density values increased with the number of cells. There was no statistically significant difference between labeled and unlabeled BMSCs (P>0.05) 0–5 days following cell labeling (Fig. 3F). At the same time interval, the cell proliferation ratio in the experimental and control groups reduced gradually with prolongation of survival time, but remained >95%.

### In vitro MRI studies of BMSCs labeled with gadodiamide

**Figure 4:**
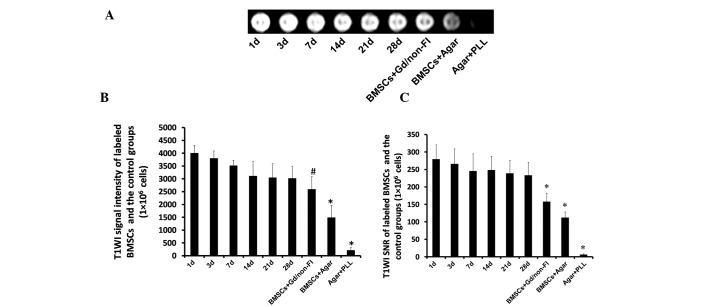
MRI signal intensity and durability at different time-points in labeled cells. (A) A total of 1×10^6^ cells were detected by clinical MRI. The T1WI signal and relevant SNR of BMSCs were observed to be attenuated with progressing cell division and proliferation. (B and C) The MRI-derived quantitative T1WI signal intensity and SNR data of labeled BMSCs increased significantly from 1–28 days compared with that in non-labeled BMSCs (BMSCs + Agar) (^*^P<0.05). The MRI signal intensity in the labeled groups at 1, 3 and 7 days subsequent was higher than that in BMSCs with Gd but non-FI (BMSCs + Gd/non-FI) (^#^P<0.05). MRI, magnetic resonance imaging; T1WI, T1 weighted image; SNR, signal-to-noise ratio; BMSCs, bone marrow stromal cells; Gd/FI, gadodiamide/FI-Arrest In reagent; PLL, poly lysine.

BMSCs labeled with the gadodiamide-FI Arrest In complex (Gd + FI) particles at different time-points were scanned using an MRI detector with spin echo *in vitro*. The minimal number of detectable cells was 5×10^4^. The results demonstrated that at 1, 3, 7, 14, 21 and 28 days following cell labeling, the signal was enhanced as shown by increased T1WI and SNR compared with those in the control groups. The T1WI and relevant SNR of BMSCs were attenuated with cell division and proliferation (Fig. 4A). Significant differences were observed between the control (BMSCs + Agar) and experimental groups on days 1–28 with regard to T1WI signal enhancement and SNR (P<0.05; Fig. 4B and C; Table I). MRI signal intensity in the labeled groups at 1, 3 and 7 days subsequent to cell labeling were higher than those of BMSCs treated with gadodiamide but without FI Arrest In reagent (BMSCs + Gd/non-FI; P<0.05; Fig. 4B). Among the labeled cells, signal intensity slightly decreased with time (days 1–28; however, these differences were not statistically significant (Fig. 4B–C).

## Discussion

In order to use stem cells for the treatment of disease, their molecular biological characteristics, gene expression profiles and signaling pathways are required to be studied and methods of stem cell modification need to be developed; furthermore, stem cell tracing studies are required to investigate the cellular migration in the body (30,31). The purpose of the present study was to investigate a novel and safe cell labeling strategy for a basic MRI contrast agent in order to provide access to a novel clinical MRI tracking method of BMSCs.

Superparamagnetic iron oxide and ultra small super-paramagnetic iron oxide predominantly shortens the T2 and T2^*^ relaxation time (32–34). Using conventional MRI contrast agents, it is difficult to distinguish stem cells from air, post-operative bleeding and iron signals originating from red blood cells (35,36). These effects lead to distortion and misinterpretation of MRI tracing of stem cells. In the clinic, as opposed to iron-based contrast agents, cellular labeling with gadolinium chelates is more permanent, and their use is safer and more economical (37,38). It is physically and chemically stable (39), and is easily dissolved in water but is not ionized. Gadodiamide can be filtrated and eliminated through the glomerulus quickly; thus, it does not accumulate to a toxic level *in vivo* (40). Gadodiamide has advantages over other particles due to its non-ionic characteristics, low osmotic pressure and high lethal dose (26,41). More importantly, its efficacy, safety and tolerance are similar or even superior to those of Gd-DTPA, which was confirmed by animal and human clinical trials in previous studies (41–44). In 1996, gadodiamide was first described to be a safe and effective marker of the nervous system of children (45). Several studies indicated that gadodiamide had a relatively low thermodynamic stability constant and conditional stability constant *in vitro* (46–48). However, as these studies were conducted *in vitro*, they did not effectively reflect physiological conditions. Another study assessed the gadolinium concentration in organs of rats following gadodiamide injection; however, this did not give sufficient information on the separation degree between gadolinium and ligand *in vivo* (49). The FDA has approved the use of gadodiamide/Omniscan for the enhancment of MRI images of human tissues and organs *in vivo*, particularly for cranial, spinal and peripheral nerves (50). However, to date, only few studies have focussed on stem cell labeling with non-ionic gadolinium, including gadodiamide (44,51).

In the present study, fluorescent Arrest-In transfection reagent was used to form a composite with gadodiamide as a novel method for cell labeling. FI Arrest-In was originally formulated for short hairpin RNA transfection and is a polyethylenimine-based lipopolymeric formulation, which is combined with a rhodamine fluorescent element and transfection reagent. Through the orthogonal test, the optimized cell uptake concentration and incubating time were determined to help reduce cytotoxicity. The delivery and localization of the Gd + FI composite in the BMSCs was confirmed by flow cytometric analysis and inverted fluorescence microscopy. In addition, tracing of Gd + FI-labeled cells by MRI was successfully performed *in vitro*, achieving the goal of real-time dual imaging. Compared with previous studies (52–55), the present study used a novel cell uptake method to reduce labeling steps and the quantity of reagents required, and improve laboratory safety. In the present study, no effects of gadodiamide on the cell viability and proliferation of BMSCs were observed *in vitro*.

In the present study MRI was able to effectively detect the labeled BMSCs. The minimum number of cells able to be detected by the MRI scanner was 5×10^4^ cells, which was similar to results reported in a previous study (55); however, the dosage of MRI contrast agent was only two-thirds of that. As Gd + FI composites were located in the cytoplasm of labeled cells, the concentration of gadolinium reduced as the cells divided (56). Therefore, the MRI detection time was limited. Although signal intensity was suggested to gradually reduce with labeling time, the signal and signal-to-noise ratio were observed to be higher in the experimental cells than those in the blank cells (BMSCs + Agar), and the signal remained sufficiently high 28 days subsequent to cell labeling *in vitro*. The T1WI signal for the MSCs + Gd/non-FI groups was higher than that of the blank cells. This demonstrated that stem cells were able to absorb small amounts of contrast agent through direct phagocytosis. However, the T1WI signal and SNR of labeled cells at 1, 3 and 7 days following cell uptake was higher than those at 1 day in the MSCs + Gd/non-FI groups. This suggested that cell labeling using the FI-Arrest In reagent led to increased cellular uptake of gadodiamide as compared with direct phagocytosis of cells, leading to an enhancement of the MRI signal.

In conclusion, by combining the clinically used Gd chelate gadodiamide (Omniscan) and a rhodamine-conjugated transfection reagent to form composites, a novel, effective, practical and rapid labeling protocol for stem cells was successfully developed using BMSCs from neonatal SD rats. Those labeled cells were detected using a conventional clinical MRI system *in vitro*, providing a foundation for BMSCs tracing *in vivo* in the future.

## Figures and Tables

**Table I:** T1WI signal intensity and SNR for different times of Gd/FI-labeled BMSCs and the control groups (1×10^6^ cells).

BMSCs	T1WI	SNR
BMSCs labeled with Gd/FI composite		
1 day	4001.37±296.58	279.05±41.24
3 days	3801.28±282.33	265.58±43.20
7 days	3511.6±211.00	245.30±49.00
14 days	3105.63±564.39	248.04±39.97
21 days	3042.12±460.05	238.42±37.17
28 days	3015.55±460.05	233.00±37.56
BMSCs + Gd/non-FI (1 d)	2589.45±494.03^a^	157.53±24.09^b^
BMSCs + Agar	1489.91±471.78^b^	112.01±16.26^b^
Agar + PLL	207.18±109.36^b^	6.29±1.82^b^

Values are expressed as the mean ± standard deviation (n=3).

a P<0.05, post-labeling at 1, 3 and 7 days vs. BMSCs + Gd/non-FI at 1 d (paired t-test).

b P<0.05, labeled cells from 1–28 days vs. the control group (paired t-test). T1WI, T1 weighted image; SNR, signal-to-noise ratio; BMSCs, bone marrow stromal cells; Gd/FI, gadodiamide/FI-Arrest In reagent; Agar, 1% agarose solution; PLL, poly-lysine.
